# Preparation and Optimisation of Cross-Linked Enzyme Aggregates Using Native Isolate White Rot Fungi *Trametes versicolor* and *Fomes fomentarius* for the Decolourisation of Synthetic Dyes

**DOI:** 10.3390/ijerph15010023

**Published:** 2017-12-23

**Authors:** Martina Vršanská, Stanislava Voběrková, Ana María Jiménez Jiménez, Vladislav Strmiska, Vojtěch Adam

**Affiliations:** 1 Department of Chemistry and Biochemistry, Mendel University in Brno, Zemedelska 1, 61300 Brno, Czech Republic; mxtinka@seznam.cz (M.V.); matalovas@volny.cz (S.V.); anuskajj@hotmail.com (A.M.J.J.); vlada.strmiska@gmail.com (V.S.); 2 Central European Institute of Technology, Brno University of Technology, Technicka 3058/10, 61600 Brno, Czech Republic

**Keywords:** CLEA, enzyme immobilization, laccase, white rot fungi

## Abstract

The key to obtaining an optimum performance of an enzyme is often a question of devising a suitable enzyme and optimisation of conditions for its immobilization. In this study, laccases from the native isolates of white rot fungi *Fomes fomentarius* and/or *Trametes versicolor*, obtained from Czech forests, were used. From these, cross-linked enzyme aggregates (CLEA) were prepared and characterised when the experimental conditions were optimized. Based on the optimization steps, saturated ammonium sulphate solution (75 wt.%) was used as the precipitating agent, and different concentrations of glutaraldehyde as a cross-linking agent were investigated. CLEA aggregates formed under the optimal conditions showed higher catalytic efficiency and stabilities (thermal, pH, and storage, against denaturation) as well as high reusability compared to free laccase for both fungal strains. The best concentration of glutaraldehyde seemed to be 50 mM and higher efficiency of cross-linking was observed at a low temperature 4 °C. An insignificant increase in optimum pH for CLEA laccases with respect to free laccases for both fungi was observed. The results show that the optimum temperature for both free laccase and CLEA laccase was 35 °C for *T. versicolor* and 30 °C for *F. fomentarius*. The CLEAs retained 80% of their initial activity for *Trametes* and 74% for *Fomes* after 70 days of cultivation. Prepared cross-linked enzyme aggregates were also investigated for their decolourisation activity on malachite green, bromothymol blue, and methyl red dyes. Immobilised CLEA laccase from *Trametes versicolor* showed 95% decolourisation potential and CLEA from *Fomes fomentarius* demonstrated 90% decolourisation efficiency within 10 h for all dyes used. These results suggest that these CLEAs have promising potential in dye decolourisation.

## 1. Introduction

Species of Basidiomycetes are known to be very interesting fungi, including white rot fungi, which are considered the most promising group of microorganisms for degrading lignin due to extracellular enzyme production [1]. One of the most important ligninolytic enzymes is laccase (Lac, E.C. 1.10.3.2), which is a blue copper polyphenol oxidase containing four copper atoms per molecule in the catalytic centre and catalyses four electron reductions of oxygen to water [2]. Laccases are stable at an acidic pH and exhibit optimum activity within the pH range from three to six [3]. In addition, laccases are active over a wide range of temperatures (20–55 °C). Thermostable laccases (60–70 °C) were also purified and characterised [4]. It is not surprising that laccase production by *Trametes*, *Pleurotus*, *Lentinula*, *Pycnoporus*, *Phanerochaete*, and *Agaricus* has been widely studied [5].

It is known that production of various fungal laccases is controlled by different genes and that laccase exhibits different isoforms with a molecular weight within the range from 55 to 110 kDa [6]. The found laccase isoforms from various fungal strains are highly important because the different isoforms possess special catalytic properties [5] and provide new potential options for laccase application. However, utilisation of laccase isoforms in a wide variety of fields has been ignored due to the lack of commercial availability. Various applications of laccase in biotechnological areas have resulted in the need to expand the spectrum of laccase positive organisms and use their laccase-producing potential [7,8,9]. Because the industrial application of laccase is still hampered by a lack of long-term operational stability and the difficulty in recycling laccase, it is necessary to improve the properties of laccase, which could be partially solved by studying its various isoforms.

Enzyme immobilisation techniques are well recognised as a common way to overcome the aforementioned drawbacks. There are four principal methods for immobilization of enzymes, such as binding to a carrier by adsorption, entrapment or encapsulation in organic/inorganic polymer, covalent and cross-linking of proteins molecules [10]. Use of the carrier unavoidably leads to ‘dilution of activity’, owing to introduction of a large portion of non-catalytic ballast, which results in lower space-time yields and productivities. Furthermore, immobilization of the enzyme on the carrier often results in substantial loss of activity, especially at high enzyme loadings. Consequently, there is increasing interest in the carrier-free immobilized enzymes, such as (CLEs)—direct cross-linking of dissolved enzymes; cross-linked spray dried enzymes (CLSDs)—cross-linking of sprayed dried enzymes; cross-linked enzyme crystals (CLECs)—cross-linking of crystalline enzymes; and cross-linked enzyme aggregates (CLEAs)—cross-linking of physically aggregated enzymes [11]. CLEA can be modified with the addition of metal ions (Fe^2+^/Fe^3+^) [12], the magnetic nanoparticles [13] or the amino-functionalized magnetite nanoparticles [14] for increased the activity, mechanical and thermal stability, and better separation from reactive mixture [12]. However, not one method is ideal for all molecules or purposes considering the inherently complex nature of the protein structure [15].

Cross-linked enzyme aggregates (CLEAs) have been recently proposed as an alternative to conventional immobilisation on solid supports and to cross-linked enzyme crystals [16]. The CLEA methodology is rapid, gentle and low-cost; the preparation is simple and essentially combines a two-step process (purification and immobilisation) into a single operation. CLEAs are easily prepared from crude enzyme extract leads to keep all isoforms of laccase [17]. Moreover, CLEA combines insoluble biocatalysts with highly concentrated enzyme activity to improve the catalytic activity and create stability against thermal and chemical denaturation (organic solvents, autoproteolysis). An important benefit of CLEA technology is low production costs, due to the exclusion of an additional (expensive) carrier [18]. Concurrently, this is an excellent method for stabilising the quaternary structures of multimeric enzymes. However, CLEA is not suitable for all enzymes, a structural feature encountered with many industrially important enzymes, such as alcohol dehydrogenases, oxidases, peroxidases, and nitrile hydratases [16]. CLEA also has some inherent limitations, such as a poorly controlled particle size, low activity difficulty of reutilization, and possible denaturation or structural modification by cross-linker [16].

The majority of previous studies have focused on lignin degrading commercial enzymes of *Phanerochaete chrysosporium* and *Trametes versicolor*, their immobilization on solid supports and to cross-linked enzymes and use for delignification, detoxification and decolourisation [19,20,21].

There has been a growing interest in studying the ligninolytic enzymes of white rot fungi with the expectation of finding better ligninolytic systems from native isolates. To our knowledge, few studies have been published dealing with CLEA immobilisation of laccase produced by native fungal isolates, and no previous study has been published on the CLEA immobilisation of laccase produced by *Fomes fomentarius*. Hence, the objective of the present work was, firstly, to investigate laccase production by native white rot fungal isolate *F. fomentarius* observed from the Czech forest. *T. versicolor* was used as a comparative strain, as *Trametes* is known as a significant producer of ligninolytic enzymes. The second objective was to improve the laccase stability of these strains by optimising the CLEA immobilisation method. Biochemical properties investigated in this study included pH optimum, pH stability, temperature optimum, thermal stability, storage, operation stability, stability against denaturation, and kinetic parameters. The final objective of this study was to test the potential application of the immobilized laccases for the decolourisation of synthetic dyes, which often have negative impacts on the environment [22,23,24,25].

## 2. Materials and Methods

### 2.1. Fungal Strains and Laccase Production

Two native fungal strains *T. versicolor* (TV) and *F. fomentarius* (FF) were isolated from trees in a Czech forest. *T. versicolor* was isolated from the dead tree trunk of *Alnus glutinosa* (49°29′34.40″ S, 16°64′47.95″ W) and *F. fomentarius* from *Fagus sylvatica* (49°17′37.02″ S, 16°38′40.02″ W).

The strains were microscopically identified and preserved on potato dextrose agar (PDA) at 4 °C. Prior to the experiment, the cultures TV and FF were grown on PDA for 10 days at 22 °C. After this, 1 × 1 cm^2^ plugs were cut and added into Erlenmeyer flasks containing 90 mL of the potato dextrose broth (PDB) pH 5.1. Copper sulphate (0.5 mM CuSO_4_·5H_2_O) was used as an inducer of laccase activity. The flasks were incubated in a shaker (150 rpm, 28 °C) and crude culture supernatant was taken after 7 days of cultivation and used for immobilisation [26]. After 7 days of cultivation, specific activity of free laccase from *T. versicolor* was 358 U/mg and 405 U/mg for CLEA laccase. Specific activity for *F. fomentarius* was 264 U/mg of free laccase and 332 U/mg of CLEA laccase.

### 2.2. Optimisation of CLEAs Preparation Conditions

Ammonium sulphate (75 wt.% saturation) was added to the crude culture supernatant in the beakers and pH of the solution was adjusted to the desired pH (6 for TV and 7 for FF, according to a previous study [27]. After continuous stirring for 0, 30, 60, 90, 120, and 150 min at 4 °C, the precipitates were recovered by centrifugation (4500 rpm/4 °C/15 min). The precipitants were collected and dissolved in the 0.1 M sodium-acetate buffer (pH 4.5), centrifuged (10,000 rpm/4 °C/5 min). The precipitation was followed by the cross-linking by using glutaraldehyde. This bifunctional reagent responds with reactive NH_2_ groups (mainly Lys) on the protein surface [11]. Glutaraldehyde is widely used as the cross-linking agent [28]. Varying concentrations of glutaraldehyde (2, 10, 20, 50, 100 mM) were used (Table 1) [10,29,30]. The solution with glutaraldehyde was kept at a low rate of stirring for different times (30, 60, 90, 120, 150 min) at 4 °C/20 °C to obtain cross-linked aggregates. Subsequently, the laccase CLEAs were centrifuged (10,000 rpm/4 °C/5 min) and washed repeatedly with buffer until no laccase activity was observed in the supernatant.

### 2.3. Laccase Activity and Protein Assay

Laccase activity of free enzymes was determined spectrophotometrically using a UV/VIS Lambda 25 Spectrophotometer (PerkinElmer, Schwerzenbach, Switzerland). Laccase activity was measured at 415 nm by detecting the oxidation of 10 mM 2,2-azino-bis-[3-ethyltiazoline-6-sulfonate] (ABTS, Sigma Aldrich, St. Louis, MO, USA, ε = 36.0 mM^−1^·cm^−1^) [31]. The reaction was carried out by pipetting 900 μL of buffer (0.1 M sodium acetate buffer pH 4.5), 50 μL supernatant/5 mg CLEA and 50 μL substrate. One unit of enzyme activity was defined as 1 μmol of substrate oxidised per min under the assay conditions. The enzyme activity assay was always performed in triplicate. The enzyme activity is expressed as specific activity in U/mg for free and immobilised laccase. The enzyme activity, specific activity, relative activity, and residual activity of free and immobilised laccase are defined by the following Equations (1)–(4):

Enzyme activity (U/mL) = [(AV)/(tεl)]/V_e_ × 1000 (1)

Specific activity (U/mg) = (1/Protein concentration) × Enzyme activity(2)

Relative activity (%) = (A_i_/A_f_) × 100 (3)

Residual activity (%) = (Final activity/Initial activity) × 100(4)

where A is the absorbance at 415 nm, V is the reaction volume, t is the reaction time, ε is the molar extinction coefficient for the ABTS oxidation, l is length of the cuvette, V_e_ is enzyme volume, A_i_ is the activity of the immobilised laccase, and A_f_ is the activity of the free laccase [32].

The protein concentration was determined according to the Bradford method [33] with bovine serum albumin as a standard. The protein concentration was always performed in triplicate.

### 2.4. pH Optimum and Stability of Free and Immobilised Laccase

The optimum pH of the free and immobilised laccases was determined spectrophotometrically using a UV/VIS Lambda 25 Spectrophotometer (PerkinElmer) with ABTS (10 mM) as a substrate using different buffers (2.2–2.4 potassium hydrogen phthalate-hydrochloric acid buffer; 2.6–7.0 citrate phosphate buffer; 7.2–8.0 phosphate buffer). Laccase stability at various pH levels (2.2–8.0) was evaluated by pre-incubating the free and immobilised laccase in appropriate buffers for 12 h. Experiments for optimum pH and pH stability were performed in triplicate and the results were given in relative form with the highest value being 100% activity [34,35].

### 2.5. Optimal Temperature and Thermal Stability of Free and Immobilised Laccase

The optimal temperature for free and immobilised laccase was determined spectrophotometrically using a UV/VIS Lambda 25 Spectrophotometer (PerkinElmer) to be between 25 °C and 60 °C in 0.1 M sodium acetate buffer (pH 4.5), where the highest activity was measured. To investigate the thermal stability, the free and immobilised laccases were incubated in a buffer, where the highest activity was determined in 0.1 M sodium acetate buffer (pH 4.5) at various temperatures (25–60 °C) for 12 h. The results for optimal temperature were given in relative form with the highest value being 100% activity. The experiment was performed in triplicate [34,35].

### 2.6. Storage and Operational Stability of Free and Immobilised Laccase

Storage stability was observed for 70 days, when free and immobilised laccase were incubated in 0.1 M sodium acetate buffer (pH 4.5) in a fridge (4 °C), and protein amount and residual activity were measured every week. Results were specified as a residual activity with the initial value being 100% activity. The experiment was performed in triplicate [36].

To evaluate the operational stability, immobilised laccases were reacted with ABTS (0.1 M sodium acetate buffer, pH 4.5) at 20 °C. Before using it in the next cycle, the immobilised CLEA was washed with 0.1 M sodium acetate buffer. This procedure was repeated for 12 consecutive reaction cycles for *Trametes* and 10 for *Fomes*, and residual activity was determined. All experiments were performed in triplicate and laccase activity in the first cycle was defined as 100% [37].

Storage and operation stabilities were determined spectrophotometrically using a UV/VIS Lambda 25 Spectrophotometer (PerkinElmer).

### 2.7. Stability of Free and Immobilised Laccase against Denaturation

Different denaturation agents (CaCl_2_, CoCl_2_, ZnCl_2_, EDTA) at a concentration of 10 μM and hydrophilic organic solvents (acetone, methanol) at a concentration of 25 vol % in 0.1 M sodium acetate buffer pH 4.5 were used to measure the stability of free and CLEAs laccases against denaturation. The mixture was incubated for 1 h at 20 °C and then the laccase activity was measured using ABTS as the substrate as described above. Protein amount was also detected. Results were specified as a relative activity with the highest value being 100% activity. The experiment was performed in triplicate. Stability against denaturation was determined spectrophotometrically using a UV/VIS Lambda 25 Spectrophotometer (PerkinElmer).

### 2.8. Kinetic Parameters of Free and Immobilised Laccase

The Michaelis–Menten kinetic parameters K_m_ and V_max_ of free and CLEAs laccases were determined using ABTS as a substrate at various concentrations (0.05, 0.1, 0.2, 0.4, 0.5, 0.75, 1.0, 1.5, 2.0 mM). The activity for each substrate concentration was determined three times spectrophotometrically using a UV/VIS Lambda 25 Spectrophotometer (PerkinElmer). The parameter values were obtained by curve fitting the plot of reaction rate versus substrate concentrations using the Statistica software (Dell Software, Aliso Viejo, CA, USA).

### 2.9. Decolourisation Experiment

Malachite green (MG), bromothymol blue (BB) and methyl red (MR) dyes were used for the decolourisation experiment. The reaction mixtures containing 7 mg/L MG, 50 mg/L BB or 100 mg/L MR, free laccase, or CLEA laccase in a final volume of 5 mL were incubated at pH 4.5, 22 °C, without shaking, in the dark. The residual dye concentration was measured spectrophotometrically at the maximum absorption peak of the dye (615 nm-MG, 605 nm-BB, and 530 nm-MR) every 2 h up to 10 h. The residual dye concentration was calculated from measured absorbance according to the following expression: (%) = [(A_0_) − (A)/(A_0_)] × 100, where % is the decolourisation percentage obtained, A_0_ the initial absorbance, and A is the final absorbance. All experiments were performed in triplicate [36,38].

## 3. Results and Discussion

### 3.1. Optimisation of CLEA Preparation

#### 3.1.1. The Effect of Cross-Linking Time

One of the most important steps during the immobilisation procedure is the optimisation of precipitation and purification of the enzymes. This step is commonly done with salting by using ammonium sulphate, but it is necessary to set the optimum conditions for the precipitation (optimum concentration of the precipitant, optimum temperature, pH, and time of precipitation). Thus, we focused on the optimisation of the isolation procedure, particularly on precipitation of the enzymes, which would be used for immobilisation in the next step. Ammonium sulphate (75 wt.%) was used to salt the enzymes and pH was chosen based on a previous study [27]. Optimum pH for *T. versicolor* was six, and for *F. fomentarius* the value was eight. For *T. versicolor* the pH optimum for precipitation is documented about a value of four to seven [10]. Kumar et al., (2012) also showed that laccase is usually precipitated in the pH middle layer.

**Figure 1:**
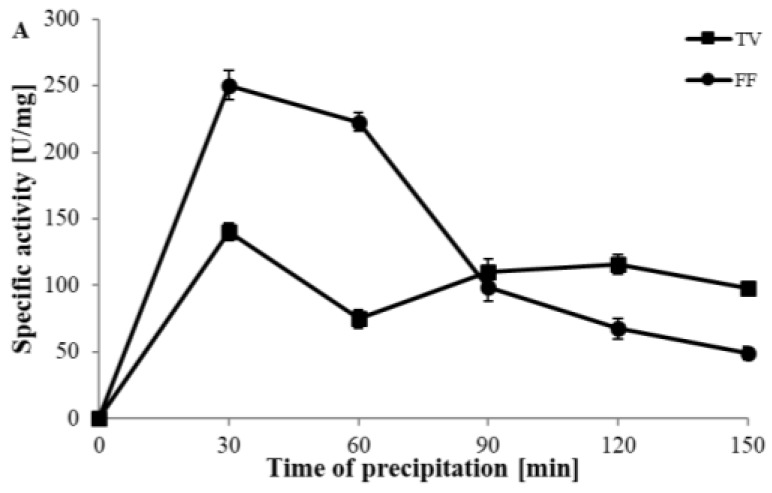

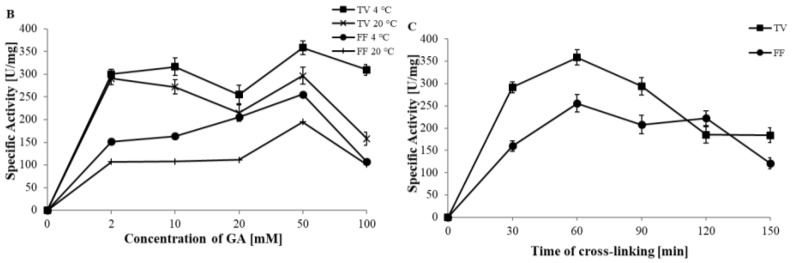
(**A**) Precipitation of laccases at different times using 75 wt.% ammonium sulphate; (**B**) Concentration and temperature of glutaraldehyde for cross-linking procedure; (**C**) Time of cross-linking using 50 mM GA and 4 °C.

Some proteins may require a longer period than 20 min of time to precipitate, so the optimal time for precipitation was determined within the range from 30 to 150 min [39]. Figure 1A shows that the highest activity for both fungi was assigned after 30 min of precipitation.

#### 3.1.2. Effects of Cross-Linking Temperature, Time, and Different Concentrations of Cross-Linking Agent

The cross-linking step is a critical step in the preparation of CLEAs because the concentration of glutaraldehyde (GA) as a cross-linking agent and cross-linking temperature and time significantly influenced the activity, stability, and particle size of the CLEA [16]. If the ratio is too low, sufficient cross-linking does not occur and CLEA cannot be formed, resulting in a CLEA that is too flexible and unstable towards leaching in water. On the other hand, too much cross-linking agent can result in a complete loss of the flexibility of laccase and hence its activity. Since each enzyme has a unique surface structure, containing varying numbers of lysine residues, the optimum ratio must be determined for each enzyme separately [40].

The influence of the amount of glutaraldehyde (2; 10; 20; 50; 100 mM) and different cross-linking temperatures (4 °C and 20 °C) on the activity is shown in Figure 1B,C. Precipitated laccases from *T. versicolor* and *F. fomentarius* were dissolved in sodium-acetate buffer pH 4.5 and used for the cross-linking procedure. For both fungi, the best concentration of GA seemed to be 50 mM, when the activity for *Trametes* was observed as 358.3 U/mg and for *Fomes* 255.7 U/mg. Higher concentrations of GA (100 mM) caused rapid loss of enzyme activity, probably due to aggregation, precipitation, and distortion of enzyme structure [41]. Similar results were showed in the work of Cabana et al. [42], who observed the reduction of the CLEA activity at GA concentrations higher than 50 mM. This result can be explained by a rigidification of the three-dimensional structure of laccase that hinders the conformational change needed for the oxidation of substrates at the active site. Our data also agrees with the study of Li et al. [43], who tested different concentrations of GA and found the best results to be between 0.5% and 1.0% (*v*/*v*) concentration of GA, which corresponds to concentration 20–50 mM GA. Opposite results were observed in a study from Kumar et al. [30], in which the highest laccase activity was detected using a lower concentration of GA (10–20 mM). It was probably caused by different preparation of CLEA. Preparation consisted of aggregation with three phases partitioning, which was formed and the interfacial layer which contained the enzyme aggregates was separated and cross-linked by GA.

The cross-linking temperature is an important factor to consider for specific activity optimisation. Our data shows that higher efficiency of cross-linking was observed at a low temperature (4 °C) compared to those prepared at 20 °C (Figure 1B). This is most likely due to thermal inactivation (denaturation) of laccase and therefore less active CLEAs [44].

### 3.2. Characterisation of CLEA Laccase

#### 3.2.1. The Influence of pH

**Figure 2:**
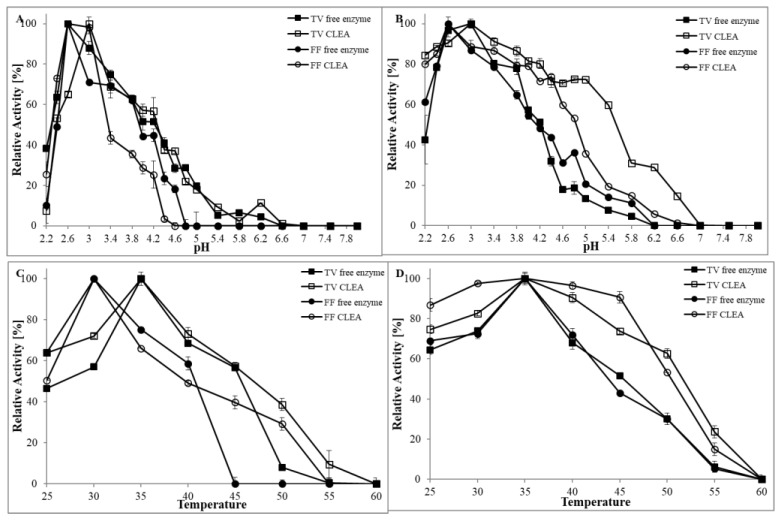
The influence of (**A**) pH optimum and (**B**) pH stability on free and CLEA immobilised laccases from *T. versicolor* and *F. fomentarius*. (**C**) The influence of optimum temperature and (**D**) thermal stability on free and CLEA immobilised laccase from *T. versicolor* and *F. fomentarius*.

The free laccase and CLEA laccase from both fungi were tested for their activity under different pH conditions from 2.2 to 8.0 (Figure 2A). The results demonstrated that *T. versicolor* had an optimal pH value at an acidic pH of 2.5 for free enzyme and 3.0 for CLEA enzyme with ABTS substrate. Moreover, it did not show any activity after pH 6.6. Therefore, it can be suggested that laccase from the *Trametes* strain belongs to a laccase that is active only in the acidic region [4]. The laccase activity at a higher pH is decreased by the binding of a hydroxide anion to the T_2_/T_3_ coppers of laccase that interrupts the internal electron transfer from T_1_ to T_2_/T_3_ centres [45]. Not only the rate of oxidation but also the reaction products can differ according to pH, whereas pH may affect abiotic follow-up reactions of primary radicals formed by laccase. Our results agree with the work of Wang et al. [46], who observed that the optimal pH of laccase from *T. versicolor* was pH 2.6, and when the pH value was higher than 6.6, all the oxidising activity towards ABTS was lost. The results for the second tested strain, *F. fomentarius*, were almost identical, as the optimum pH for free laccase was 2.6 and for CLEA 3.0, but there was no observed activity after pH 4.6. In pH values greater than 4.6, the enzyme activity decreased gradually and the enzyme was completely inactivated at a higher alkaline pH. This phenomenon can be explained by the difference in redox potential between a reducing substrate and the type 1 copper in the active site of the enzyme and the inhibition of type 3 copper by hydroxide ion at a higher pH [47]. In the work of Neifar et al. [48], *F. fomentarius* commercial laccase had an optimum pH of 4.0, but in this case, dimethoxyphenyl was used as a substrate. However, at pH values greater than 4.0, the enzyme activity also decreased gradually (Figure 2A).

This study also observed an insignificant increase in optimum pH for CLEA laccases with respect to free laccases for both fungi. It is known that the optimum pH for an immobilised enzyme shifting to a higher or lower pH depends on the type of immobilisation [49] and ionic interaction between the enzyme and cross-linking agent [50]. The shift in optimum pH towards a less acidic pH value upon immobilisation may be due to the difference in hydrogen ion formation of the glutaraldehyde during cross-linking process.

Chen et al. [21] suggested that the higher pH value for the CLEA immobilised enzyme was caused by the fact that optimum pH is based on the pK_a_ of amino-acids in the vicinity of active site of enzymes. After cross-linking, glutaraldehyde reacts reversibly with amino groups of enzymes and changes the optimum pH of the enzyme. Stability is one of the most important factors for evaluating enzyme application potential [51]. The pH stability was determined at 25 °C for 12 h in pH from 2.2 to 8.0 (Figure 2B). CLEA immobilisation showed improved pH stability for both tested strains, compared to free laccase. Relative activity from *Trametes* was higher than 50% to pH 4.2 for free laccase and 5.4 for CLEA. For the second fungus, *Fomes*, the relative activity of free laccase decreased in pH to 4.0, but CLEA was stable at 4.8. It would be expected that cross-linking of enzymes from both fungi increased the stability of the laccase by protecting reactive groups, and CLEA prepared from the laccase indeed exhibited improved stability against different pH levels [17]. An additional benefit of the CLEA technology is stabilisation of the quaternary structures of multimeric enzymes, a structural feature often encountered with redox metalloenzymes. Our results are in agreement with the study of Zhu et al. [52], who tested immobilised commercial laccase and showed a higher resistance to changes in the pH value of the buffers.

#### 3.2.2. The Influence of Temperature

It was reported that during enzyme immobilisation, free movement of enzyme molecules was obstructed, even at higher temperatures. Thus, enzyme denaturation was not observed due to the protection of amino acids at the active site. The optimal temperature for free and immobilised laccase was tested between 25 °C and 60 °C and was studied in the pH of the buffer, where the highest activity was observed (citrate phosphate buffer, pH 2.6). Free laccase and CLEA presented similar trends in regard to the effect of temperature for both fungi. The results showed that the optimum temperature for both free laccase and CLEA laccase was 35 °C for *T. versicolor* and 30 °C for *F. fomentarius*, followed by a stepwise decrease in activity with the increase of temperature (Figure 2C). Moreover, we did not detect any activity at 60 °C, where complete inactivation of free and CLEA laccases of *Trametes* occurred. Considering *Fomes* laccase, the situation was different, as the free enzyme was inactivated at 45 °C, but CLEA at 55 °C. Many authors (Irshad et al., 2011; Zhu et al., 2007) report the optimum temperature of laccase to be 30–50 °C, which agrees with our study. Our observations showed that CLEA immobilisation of laccase did not have a significant influence on pH and optimum temperature.

The thermal stabilities of the free and CLEA laccases were also investigated (Figure 2D). Both CLEA immobilised laccases showed significant improvement of thermal stability in comparison to free laccase. CLEA laccases from *Trametes* and *Fomes* retained more than 50% of their activity at 50 °C, whereas free laccases rapidly decreased in activity after 40 °C. Many authors have observed that the CLEAs are stable over a wide pH and temperature range [42,53,54] in comparison to free laccase. It is known that the thermal stability of the immobilised laccase is significantly improved [55]. This enhancement of thermal stability of CLEAs could be due to the covalent cross-linking among enzyme aggregates [30,50]. This means that the laccase immobilised by cross-linking could increase the resistance of high catalytic activity within a broader temperature range, and this property is crucial in practical applications, e.g., in the treatment of textile industrial waste effluent [21].

#### 3.2.3. Storage Stability

The economics of industrial enzyme bioprocesses are influenced by the enzyme production cost, which accounts for more than half of the overall production cost. Therefore, long-term storage of industrial-value-added enzymes without a decrease in biological activity has attracted considerable attention in recent years [56]. Storage stability is an imperative advantage of CLEAs over free enzymes because free enzymes can lose their activities fairly quickly [20].

**Figure 3:**
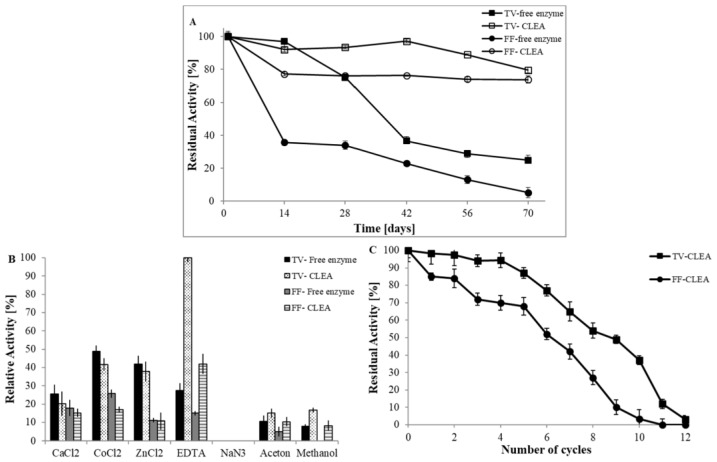
(**A**) Storage stability of free enzyme and CLEA from *T. versicolor* and *F. fomentarius*; (**B**) The relative activity of the free and immobilised laccases incubated with chemical denaturants; (**C**) Operational stability of CLEAs from *T. versicolor* and *F. fomentarius*.

To investigate the effect of immobilisation on the storage stabilities of laccase, both free enzymes and CLEAs were stored at 4 °C. After 70 days, the CLEAs retained 80% of their initial activity for *Trametes* and 74% for *Fomes*, respectively, and free laccases 25% and 5% of their initial activity for *Trametes* and for *Fomes* (Figure 3A). The activity of free laccases for both fungi dropped significantly more quickly than that of the immobilised laccases under the same storage conditions. After 70 days of storage, only 5% of the relative activity of free laccase from *Fomes* was observed. Free laccase from *Trametes* lost more than 70% of activity. This could be caused by the structural denaturation of the laccase [57], which is more significant in cases of free enzymes in comparison to CLEA laccases. As the number of storage days increased, the immobilised laccases of both fungi exhibited higher stability, which can be attributed to the limited conformational changes in enzyme molecules in the cross-linked matrix [58]. In a study from Matijošytė et al. [10], similar results were observed. CLEAs exhibited the expected increase in storage stability compared to the free enzyme after 40 days of storage.

#### 3.2.4. Stability against Denaturing Conditions

Immobilisation generally results in laccase stabilisation against chemical denaturation, which was also observed in our study (Figure 3B). In the presence of EDTA (10 µM) and 25% (*v*/*v*) methanol and acetone, improvement in stability of CLEA in comparison to free laccases was determined. The formation of CLEAs stabilises the enzyme in the presence of organic solvents [59] due to the rigidity of the biocatalyst formed. EDTA may inhibit metalloenzymes like laccase by binding irreversibly to the metal ions at the active site. This stabilisation could be due to hindering mass transfer of EDTA into the CLEA amorphous structure. Surprisingly, the prepared CLEAs enzymes became more resistant to this reagent and obviously improved stability against EDTA. With respect to NaN_3_, no activity was observed for both forms of laccase. Laccase in the form of CLEA was also less stable against a 10 µM solution of the inorganic salts ZnCl_2_, CoCl_2_, or CaCl_2_ than free laccase. This could be caused by the fact that the small dimension of the ions allowed them to easily migrate into the structure of the CLEAs and inactivate the laccase to an equal extent. Nonetheless, the low concentrations used could explain the salts’ low impact.

#### 3.2.5. Kinetic Parameters of Free and Immobilised Laccase

The kinetic parameters (K_m_, V_max_) of free and CLEA laccases were determined using various concentrations of ABTS as substrate. Kinetic testing of free laccase and the CLEA from *T. versicolor* revealed that K_m_ value after immobilisation of the enzyme decreased (from 1.9 mM to 0.8 mM respectively) (Table 2). The relationship between a rate of reaction and the concentration of substrate depends on the affinity of the enzyme for its substrate expressed as K_m_. The lower value of K_m_ for CLEA laccase indicated a higher affinity of the CLEA laccase for the ABTS. It implies that interaction between enzyme and substrate may have been strengthened by a suitable orientation of the enzyme active site towards the substrate [60]. On the other hand, the kinetic behaviour of K_m_ for laccase from *F. fomentarius* was increased via CLEA immobilisation (from 0.087 mM to 0.39 mM). This can be explained by the increased flexibility of enzyme molecules after being cross-linked and bonded with GA. It means that the substrate has not undergone diffusional limitation with respect to CLEAs due to insolubilisation. This is unlike conventional immobilisation techniques, wherein any change in the conformation of the insolubilised enzyme induces steric hindrance or alters the active site of the enzyme. Increased K_m_ value for immobilised laccase has been reported in previous immobilisation studies [61].

The higher V_max_ associated with CLEA laccases for both fungi may be attributed to the highly active enzyme obtained from precipitation followed by cross-linking the protein with GA (Table 2). Enzyme immobilisation does not ensure that enzyme molecules are attached in their correct conformation (i.e., involving residues away from active site), which strongly affects the V_max_ of the enzyme. In the case of an internal diffusion barrier, the enzyme is in a cross-linked aggregate formation, which limits diffusion of substrate molecules into the structure, which then affects K_m_ followed by V_max_.

#### 3.2.6. Operational Stability of Immobilised Laccase

Operational stability is an important reason for immobilising cost enzymes, which facilitates their recovery from the reaction mixture, resulting in the simplification of downstream processing. It is important to determine the retained activity of a batch of immobilised enzymes after each cycle of reaction. From the data, an optimum number of cycles that can be used from each batch of immobilised laccase enzymes is determined. This result is important to industries as it affects the production cost.

We subsequently studied how many cycles were needed for the activity to drop to zero. CLEA laccase from *Trametes* was used for 12 cycles, which after the eighth cycle lost 50% of original activity. In comparison, CLEA laccase from *Fomes* was used for 10 cycles, and after sixth cycle, the activity was less than 50% (Figure 3C). We can postulate that these immobilised enzymes can be reused for more than 10 cycles, as long as the retained activity is still applicable for an economical production. In the work of Kunamneni et al. [36], the immobilised laccase also showed good operational stability, maintaining 84% of its initial activity after 17 cycles of oxidation of ABTS. In the study of Sinirlioglu et al. [38], CLEA laccase reusability was studied with five cycles. After the fifth cycle, the activity of CLEA laccase decreased to nearly 60% of its original activity. According to these and our own results, CLEA laccase has potential use in industrial applications.

#### 3.2.7. Decolourisation Experiment

**Figure 4:**
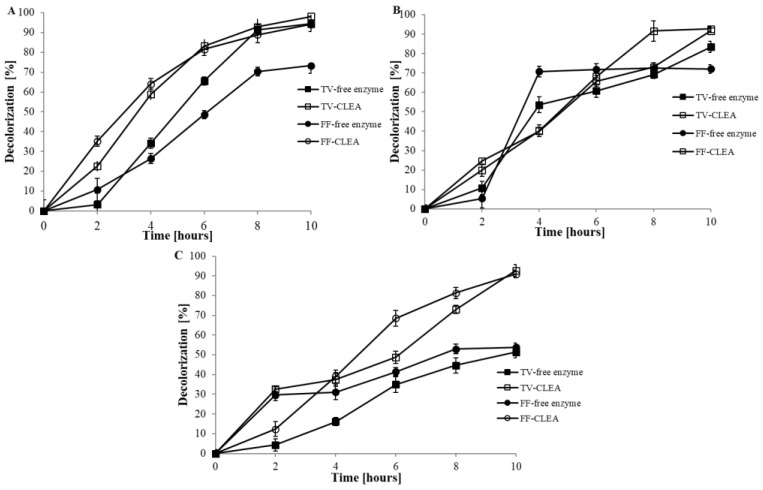
Experiment of decolourisation ability of CLEA and free laccases from *T. versicolor* and *F. fomentarius* for (**A**) Malachite Green; (**B**) Bromothymol Blue, and (**C**) Methyl Red.

The decolourisation capacity of various structurally distinct dyes by free and CLEA laccases from *T. versicolor* and *F. fomentarius* was monitored by the decrease in absorbance at the maximum absorption wavelength of each dye. Tested dyes, malachite green (MG), bromothymol blue (BB), and methyl red (MR), were selected based on a previous study [26]. The ability to decolourise dyes by CLEA laccases from *T. versicolor* and *F. fomentarius* is illustrated in Figure 4A–C. In the case of free laccases, the highest decolourisation of around 70–80% was achieved for MG and BB after 10 h of incubation and around 50% for azo dye MR. It is like that this was caused by the fact that MG and BB share a very close structure with three benzene rings, and these dyes may fit well with the enzyme activity centres and can be degraded [62]. The ability to decolourise MR, which belongs to azo dyes, was around 50% after 10 h, which agrees with work of Sharma et al. [63]. Both fungi displayed very high ability to decolourise different synthetic dyes.

Many authors have observed that immobilised laccase usually has a higher capacity of dye decolourisation than free laccase [64] . This is in agreement with our study, where higher ability to decolourise was shown for CLEA laccases at around 95% for *T. versicolor* and 90% for *F. fomentarius* for all used dyes after 10 h of incubation. Our data agrees with the study of Sinirlioglu et al. [38], who found that after 24 h, laccase and CLEA laccase decolourisation efficiency was nearly 90%. Observed data implied that dye affinity is different for different enzymatic complexes, underlining the influence of immobilisation on the laccase behaviour [65].

## 4. Conclusions

The immobilised biocatalysts displayed improved thermal and storage stability paired with good performance for reusability. The results were comparable to commercial enzymes; therefore, the use of native isolates can significantly reduce the cost of immobilised preparations. Based on this, CLEA prepared from ligninolytic laccases from native isolates seems a promising and cheap method for immobilisation due to the satisfactory performance of the immobilised biocatalyst. Application of CLEA immobilised laccase to decolourise synthetic dyes could spread by using this method in the food industry, bioremediation, biofuel production, cosmetics, and biomedical science, in addition to nanobiotechnology applications in batch and bioreactors.

## Figures and Tables

**Table 1:** Cross-linked enzyme aggregate (CLEA) preparation conditions.

Concentration of glutaraldehyde (GA) (mM)	2	10	20	50	100
Time of cross-linking (min)	30	60	90	120	150
Temperature of cross-linking (°C)	4	20			

**Table 2:** Kinetic parameters of free and CLEA laccases.

	K_m_ Free Laccase (mM)	K_m_ CLEA Laccase (mM)	V_max_ Free Laccase (U/mg)	V_max_ CLEA Laccase (U/mg)
*T. versicolor*	1.9	0.8	0.027	0.3
*F. fomentarius*	0.087	0.39	0.029	0.4
